# A Review of Current Systems, Materials, and Protocols for 3D‐Printed Splints, Crowns, and Dentures

**DOI:** 10.1111/jerd.70125

**Published:** 2026-02-17

**Authors:** Pouya Sabanik, Ting‐Chia Liu, Mahmoudreza Tabatabaeian, Mateus G. Rocha, Neeraj Surathu, Nathaniel C. Lawson

**Affiliations:** ^1^ Department of Clinical and Community Sciences The University of Alabama at Birmingham School of Dentistry Birmingham Alabama USA; ^2^ Department of Restorative Dental Sciences University of Florida School of Dentistry Gainesville Florida USA; ^3^ The ACE Institute Hamilton New Zealand

**Keywords:** 3D printing, bonding protocols, dental crowns, filler content, occlusal splints, postcuring, printed dentures, SLA, vat polymerization, zirconia

## Abstract

**Objectives:**

To summarize and synthesize evidence‐based conclusions on optimal three‐dimensional (3D) printing technologies, materials, and clinical protocols for splints, crowns, and dentures.

**Overview:**

In resin vat printing, stereolithography (SLA) offers superior surface finish, while liquid crystal display (LCD) and digital light processing (DLP) excel in speed for larger prints, with LCD providing a cost advantage. Ceramic vat printing yields clinically acceptable strength but is equipment‐ and time‐intensive. Optimal angulation varies by application: splints (0°), crowns (150°–210°), dentures (45°–90°). A 50 μm layer height is preferred for most restorations, with dentures tolerating 100 μm. Commonly used resins employ low‐viscosity monomers (e.g., ethoxylated bisphenol A dimethacrylate [Bis‐EMA]) that limit stiffness and fast photoinitiators (e.g., ethyl (2,4,6‐trimethylbenzoyl) phenylphosphinate [TPO]), requiring 385–405 nm curing.

**Conclusions:**

Flexible splints excel in fracture toughness and impact strength; firm splints match milled wear performance. Printed crowns, while weaker than milled options, offer toughness; wear rates remain a concern. Printed denture bases vary in strength but can match or exceed conventional options; printed teeth show favorable wear resistance. Postprocessing, polishing, and bonding protocols critically influence outcomes.

**Clinical Significance:**

Employing validated printing parameters, selecting the appropriate resin, and following evidence‐based protocols allows clinicians to maximize restoration accuracy and mechanical performance while leveraging 3D printing's efficiency and cost benefits.

## 1 Introduction

Three‐dimensional (3D) printing has become increasingly prevalent in dental practice and laboratory workflows. A 2022 survey of dentists in the United States reported that 17% had a 3D printer in their office, while approximately 62% of dental laboratories had adopted 3D printing by the same year [1, 2]. Among dentists, the most commonly reported uses include models (62%), splints and occlusal devices (50%), surgical guides (48%), temporary crowns and bridges (36%), definitive crowns and bridges (12%), and dentures (14%) [2]. Dental laboratories report similar trends, with 3D printing most frequently used for model fabrication (75%), followed by splints (56%), temporary restorations (48%), surgical guides (46%), impression trays (37%), and dentures (31%) [1]. To provide a focused and clinically meaningful scope, this review does not address applications such as models, surgical guides, and impression trays, which are primarily used for short‐term or indirect clinical purposes. Instead, this review focuses on the most‐commonly used oral prostheses and appliances intended for extended intraoral service, including splints, crowns, and dentures. These applications place greater demands on material properties and therefore warrant closer examination.

One of the primary drivers of interest in 3D printing for these applications is its potential economic and efficiency advantages relative to subtractive manufacturing. Unlike milling units, which are limited to producing a single restoration at a time, 3D printers can fabricate multiple prostheses simultaneously [3, 4]. In addition, 3D printers are substantially less expensive than milling machines (approximately seven times lower in initial cost), do not incur recurring expenses such as bur replacement, and use relatively low‐cost consumable materials [3].

The available evidence indicates that 3D printing is a viable method for producing clinically acceptable dental prostheses and appliances. Recent review articles have reported that the internal fit, marginal fit, and trueness of 3D‐printed fixed restorations are generally comparable to those of milled restorations across ceramic and resin‐based materials, although differences have been observed depending on material type and measurement location [5, 6, 7]. With respect to complete dentures, their occlusal fit, intaglio accuracy, and cameo accuracy were reported to be inferior to milled dentures but similar or better than conventional dentures [8, 9]. Importantly, these review articles consistently indicate that the performance of 3D‐printed materials is highly dependent on the specific conditions under which they are fabricated, including the printing technology, material formulation, and postprocessing workflow [5, 6, 8].

Therefore, the purpose of this narrative review is to synthesize the current evidence on the technologies, materials, and workflows used in the 3D printing of splints, crowns, and dentures. This review aims to identify and critically evaluate the most relevant laboratory and clinical studies in this field and to provide practical, evidence‐informed clinical recommendations, while acknowledging that 3D printing remains an emerging technology and that the certainty of clinical protocols is constrained by the limited body of available evidence.

## 2 Printing Technology

The most commonly used printing technologies in dentistry include vat polymerization, material jetting (discussed later), direct metal laser sintering (outside scope of this review), and fused deposition modeling (FDM) [10]. Although FDM may be used to fabricate models [11], the long printing time, porosity, and lack of biocompatible resins make it an unsuitable technology for oral prostheses [12]. A comparison of the different printing technologies is presented in Table 1.

**TABLE 1:** Clinical advantages and disadvantages of different printing technologies.

Printing technology	Advantage	Disadvantage
Stereolithography (SLA)	Less pixelated and smoother prints than DLP and LCD [13, 14].	Print time tied to the size of the layer [12].
Digital light processing (DLP)	Less pixel shadowing and light bleeding than with LCD printers [12] which may produce higher surface quality.	High cost of DMD component
Liquid crystal display (LCD) masking	Lower cost due to low cost of LCD panels.	LCD screen may require replacement over time
Digital press stereolithography (DPS)	Allows printing of more viscous resins [15], minimizes resin handling, and increases printing speed.	Resin must be purchased in capsule which increases cost, the 15 mm diameter build platform limits the size of printed objects
Ceramic printing	Allows printing of high strength ceramics.	Long debinding time makes process time‐intensive

### 2.1 Printers for Resin and Resin Composites Materials

Printing of most resin and resin composite materials is currently accomplished with vat polymerization. Vat polymerization involves a tank of liquid resin in which objects are formed through layer‐by‐layer curing using a light source. This category includes stereolithography (SLA), digital light processing (DLP), liquid crystal display (LCD) masking, and digital press stereolithography (DPS) technologies [15].

SLA printers (e.g., Form 3B, Formlabs, Somerville, MA, USA) use a UV laser that precisely traces and cures each layer of liquid photopolymer resin on the surface of a resin vat. The laser beam is directed by galvanometer‐driven mirrors (galvos), and the resolution of these printers is determined by the movement of the galvanometer and the spot size of the laser [12]. Objects printed near the edges of the build platform exhibit lower accuracy, possibly due to variations in light exposure with longer distances of light travel [16].

DLP printers (e.g., Sprintray Pro 55S and 95S, Sprintray, Los Angeles, CA, USA; NextDent 5100, 3D System, Rock Hill, SC, USA) cure resin using a digital projector that flashes an entire image of a layer at once onto the resin vat. The projected light is controlled by a digital micromirror device (DMD, an array of millions of microscopic mirrors), allowing for fast curing of full layers rather than point‐by‐point scanning [17]. As each layer is exposed simultaneously, DLP printing is typically faster than SLA. It is advantageous for DLP printers to employ 405 nm UV light as DMDs deliver more power with 400–420 nm light than 363–400 nm light [17].

LCD printers (e.g., Ackuretta Sol, Ackuretta, Taipei City, Taiwan; NextDent LCD1, 3D Systems; Sonic 4K 2022, Phrozen, Taipei City, Taiwan) use an LCD panel (a matrix of electrically controlled liquid crystal cells) as a mask that selectively blocks or transmits light from a backlight, typically an array of LEDs behind a polarized filter [17]. Similar to DLP, this produces a pixel‐based exposure pattern that cures an entire resin layer at once. LCD printers are generally less expensive than DLP systems because LCD panels are mass‐produced and widely available, though the panels may need periodic replacement. The resolution of an LCD printer depends on the pixel density of the screen; however, light leakage between adjacent liquid crystal cells can reduce effective resolution [12].

DPS printing (e.g., Midas, SprintRay) represents an innovative resin‐control method that has recently been applied in DLP printing with high viscosity materials [15]. It utilizes a two‐part disposable capsule system to print highly‐filled resins [15]. One chamber of the capsule holds the uncured ceramic‐filled resin, which is precisely extruded by a piston mechanism into the adjacent chamber. There, the resin is spread between a miniature build plate (15 mm diameter) and a DLP projector that cures each successive layer of the restoration [15]. This approach enables the printing of resins that are too viscous for conventional vat‐based systems, minimizes handling and maintenance of resin trays, and shortens overall printing time.

Comparing the speed and accuracy of different printing technologies can be misleading, as variations often exist between brands within the same category. For instance, when multiple brands of commercial 3D printers from each technology type were evaluated, no technology proved to be inherently more accurate than the others [18]. Some generalizations can be made regarding printing technologies. DLP light projection produces less pixel shadowing and light bleeding to the resin than is observed with LCD printers [12], which may produce higher surface quality. As SLA prints via the continuous movement of a laser rather than through an array of mirrors or crystal cells, prints with this technology may be less pixelated and smoother [13, 14]. SLA printers, however, must draw the entire image whereas DLP and LCD may project an entire image. As a result, the time required to print with SLA printers will be tied to the size of the layer (number and size of printed objects) [12].

### 2.2 Printers for Ceramic Materials

The use of fully ceramic printed materials is an emerging field in dentistry. Several additive manufacturing techniques have been explored, including binder jetting, material extrusion and jetting, and powder bed fusion [19]. Current commercial applications of printed dental ceramics, however, rely primarily on vat photopolymerization. Some manufacturers use top‐down SLA‐based systems (e.g., Ceramaker 100, 3DCeram, Limoges, France), while others employ a DLP‐derived process known as lithography‐based ceramic manufacturing (LCM, e.g., CeraFab, Lithoz, Vienna, Austria) [20]. A recent study reported higher strength in zirconia produced with an SLA compared to an LCM printer, though the difference may have been attributable to variations in print orientation [20, 21].

In ceramic vat printing, the build platform descends into a slurry of finely milled ceramic particles dispersed in a photosensitive resin which is photopolymerized to form a “green body” containing approximately 86 wt% ceramic particles in a resin matrix [22]. This is subsequently converted into a dense “brown body” through debinding (binder removal at ~487°C) followed by sintering (ceramic particle fusion at ~1500°C) [22]. To minimize cracking and porosity, debinding is typically performed in two stages: vacuum or nitrogen debinding to reduce polymer decomposition rate, and air debinding to eliminate residual carbon [23]. While effective, the process is time‐intensive, taking up to several days, limiting commercial application. Sintering then follows, accompanied by 22%–25% shrinkage, which must be accounted for during digital design [22].

## 3 Technical Considerations When Printing

An important consideration in 3D printing for clinical applications is that the materials must be validated. Validation is not only about confirming that a *materia*l has suitable properties for safe intraoral use, but also about ensuring that the *entire system*, including printing and postprocessing parameters, produces consistent and acceptable outcomes for that material [10]. When a resin is validated for a specific printer, a corresponding profile of printing parameters is established. These typically include the build platform speed, the exposure time for each layer, and the lift distance that allows resin to refill beneath the platform [24]. Together, these settings ensure the resin is adequately polymerized, that fresh resin adequately refills the gap beneath the build plate to prevent voids, and that the printed object does not distort or detach during peeling from the vat window [2, 17, 25]. Often these parameters vary depending on which region of the object is being printed (i.e., longer exposure and slower speed for the initial layers) [24]. Other parameters can be adjusted by the operator to balance total printing time, ease of support removal, and the properties of the final part. These include the printing angle, the placement and density of supports, and the chosen layer thickness. Recommended parameters to optimize properties are presented in Table 2.

**TABLE 2:** Recommended 3D printing parameters for 3D‐printed splints, crowns, and dentures.

Application	Recommended build orientation	Suggested layer thickness	Support strategy	Primary rationale	Key trade‐offs
Splints	0° (intaglio parallel and facing away from build plate) [26]	50 μm [27]		Maximizes accuracy [26]	Requires maximum amount of support removal, 90° allows slightly greater strength [27]
Crowns	150°–210° (inverted, intaglio facing away from build plate) [28]	50 μm [29, 30, 31, 32, 33, 34]	Light, thin supports on external surfaces [35]	Maximizes accuracy [30, 31, 32, 33, 35]; improves strength [29, 34]	
Denture bases	45° (intaglio facing build plate) or 90° [36]	50 μm [37, 38, 39] (100 μm acceptable when prioritizing efficiency) [40]	Supports critical on overhangs and thin areas and less critical on palate [41, 42]	Improves intaglio accuracy [36, 40]; minimizes supports	Increased printing time; slightly lower strength and higher roughness [43, 44]
Denture teeth^a^	0° (occlusal parallel and facing build plate)	50 μm	Fine supports placed on occlusal	Maximizes accuracy; improves strength; does not affect wear resistance [45]	Requires support removal on occlusal surfaces

^a^ Denture teeth resin is often the same or similar to crown resin.

### 3.1 Printing Angulation

The printing angle on the build plate, where 0° means the device's occlusal surface is parallel to the plate, affects both accuracy and mechanical strength [46, 47]. For occlusal splints, printing flat (0°) generally yields greater accuracy [26]. Differences in strength are modest; prints at 90° occasionally demonstrate marginally higher values [26]. For posterior crowns, the best marginal and internal fit is often seen at 150°, 180°, and 210°, meaning the intaglio faces away from the plate and is either parallel or tilted 30° [28]. Crown strength tends to be greatest when printed flat to the build platform (0° or 180°) [48]. In denture bases, printing at 45° or 90° maximizes accuracy and adaptability [36], while printing at 0° yields the highest flexural strength and the lowest surface roughness and *Candida albicans* adhesion [43, 44]. In denture teeth, no difference was noted in wear resistance for different printing orientations [45].

From a workflow perspective, steeper angulation (e.g., 90°) maximizes build plate utilization, but it also increases build height and thus printing time. Proper orientation minimizes the need for excessive support structures, reducing postprocessing effort and protecting critical surface details [12]. Angulation also alters the footprint of each layer on the resin vat window relative to its attachment area on the build plate. This allows angled prints to maintain strong build plate adhesion while reducing peeling forces during separation, thereby lowering the risk of print failure [12].

### 3.2 Supports

Support structures are essential in 3D printing because they stabilize overhanging features and preserve complex geometries during fabrication [49]. They also have drawbacks, as more supports increase material use, extend postprocessing time, and can leave surface defects or inaccuracies after removal [49]. Interestingly, studies evaluating the internal fit of printed crowns indicate that thinner supports are preferable [35] and that reduced support density does not adversely affect fit [50]. For denture base accuracy, placement of supports is most critical at thin areas and overhangs [41], and support density can be reduced on the palatal and border areas [42]. Because support density and diameter also influence print reliability by helping parts remain adhered to the build plate, the benefits of minimizing supports must be balanced against the risk of print failure. Adhesion to the build plate is further promoted by intentionally overexposing the first layers of a print. This can distort features at the build plate interface, often called “elephant footing,” so critical surfaces should not be printed directly on the build plate [12].

### 3.3 Layer Thickness

Layer thickness is a critical parameter in additive manufacturing, directly influencing both the efficiency of print time and the mechanical, surface, and dimensional properties of dental prostheses. Reducing layer height increases total print time but enhances polymerization between successive layers, improving interlayer bonding and, in many materials, overall strength [29, 46]. For occlusal splints, printing at 50 μm rather than 100 μm can yield slight improvements in strength [27] but does not affect wear resistance [51]. For crowns, a layer height of approximately 50 μm has been shown to optimize print trueness [30], margin quality [30], and internal and marginal gap dimensions [31, 32, 33] compared with both finer (25 μm) and coarser (100 μm) settings. Mechanical performance of crown resins also peaks at 50 μm [29, 34], with strength differences becoming more pronounced in vertically oriented builds, where tensile stresses are concentrated between layers [52]. For denture fabrication, decreasing layer height from 100 to 50 μm has been associated with modest gains in flexural strength [37] and reductions in surface roughness [38, 39], while exerting minimal influence on overall dimensional accuracy [40].

## 4 Printing Materials

### 4.1 Printed Resins

3D printing resins are typically composed of polymerizable monomers, photoinitiators, and, in some formulations, filler particles. Unlike the highly filled materials used in direct composite restorations, 3D printing resins must exhibit sufficiently low viscosity to enable efficient reflow beneath the build platform after each layer is cured [17, 53]. The resin must also be viscous enough such that it does not drip from the build plate or spread excessively when compressed by the build plate [53]. In theory, high resin viscosity can be mitigated to enable printability by reducing build‐plate speed, heating or agitating the resin tank, or mechanically redistributing the resin [53]. Aside from viscosity modification, a resin formulation must allow for controlled light transmission to ensure consistent and accurate layer‐by‐layer photopolymerization during the printing process [17]. A summary of the components of 3D printed resins and their effect on clinically relevant properties is presented in Table 3.

**TABLE 3:** Material modifications to accommodate for 3D printing.

Component	Reason for modification	Effect on clinical properties
Resins	Resin viscosity must be low enough to enable reflow beneath build platform but high enough to prevent drip from the build plate or excessive spread when compressed by the build plate [17, 53].	Use of low viscosity monomers (e.g., UDMA and Bis‐EMA) limits stiffness of printed materials [54, 55, 56, 57].
Photoinitiator	Need to use Type I phosphine oxide photoinitiators that undergo faster polymerization to accommodate rapid, layer‐by‐layer curing [58, 59].	UV light at 385 and 405 nm is required to cure these resins, as these wavelengths correspond to photoinitiator peak absorbances at 372 and 382 nm, with 385 nm providing the closer match [60]. Clinicians should be aware of potential toxicity of TPO [61].
Filler	Resin must have adequate viscosity (see above) [17, 53] and allow for controlled light transmission [17, 62].	Filler particle incorporation currently limited to ~60 wt% [54, 63] which may limit hardness, strength and stiffness [54, 63, 64].

#### 4.1.1 Resin Monomers

Bisphenol A‐glycidyl methacrylate (Bis‐GMA), the primary methacrylate used in most direct resin composites, exhibits excessively high viscosity, making it unsuitable for routine use in printable resin formulations [54, 55, 56]. Urethane dimethacrylate (UDMA) is a lower‐viscosity dimethacrylate that is commonly incorporated into commercial 3D‐printed resins [54, 55, 56]. The reduced viscosity of UDMA is attributed to a lower rotational energy barrier within its molecular backbone, which confers greater chain flexibility [54, 55]. Consequently, UDMA‐containing resins typically demonstrate a lower modulus of elasticity and increased toughness, properties that are advantageous for applications such as removable prosthetics and full‐arch appliances [54]. Additional monomers used to reduce resin viscosity include triethylene glycol dimethacrylate (TEGDMA), a low‐molecular‐weight diluent, and ethoxylated bisphenol A dimethacrylate (Bis‐EMA), a Bis‐GMA analog that lacks hydroxyl groups [55]. However, incorporation of these viscosity‐reducing monomers involves trade‐offs: TEGDMA increases polymerization shrinkage [65], while Bis‐EMA reduces strength and stiffness [55, 56, 57]. In summary, the use of lower‐viscosity monomer systems in 3D‐printed resins generally results in materials with reduced stiffness.

The exact consistuent monomers within a commercial resin are typically unknown to both clinicians and researchers. Although manufacturers are aware of the chemical compositions of their resin, only the hazardous components are required to be listed on their safety data sheets (SDS). Identification of resin components by reverse engineering (e.g., nuclear magnetic resonance, mass spectrometry, Fourier transform infrared spectroscopy) is challenging because, once the monomers are blended, individual compounds are difficult to isolate [54].

#### 4.1.2 Initiators

Camphorquinone (CQ) is the most widely used photoinitiator in direct restorative composites and functions as a Type II system that requires a tertiary amine co‐initiator to generate free radicals. Although effective for chairside restorations, CQ exhibits relatively slow polymerization kinetics, making it poorly suited for the rapid, layer‐by‐layer curing required in 3D printing [58, 59]. In contrast, Type I phosphine oxide photoinitiators used in printing resins, including ethyl (2,4,6‐trimethylbenzoyl)phenylphosphinate (TPO), its liquid analog (TPO‐L), and phenylbis (2,4,6‐trimethylbenzoyl) phosphine oxide (BAPO), undergo direct cleavage upon light exposure, resulting in faster polymerization and higher curing efficiency [58, 59]. These initiators absorb in the ultraviolet‐A (UV‐A) to violet wavelength range (TPO and TPO‐L peak absorbance at 372 nm and BAPO peak absorbance at 382 nm), which corresponds to the 385 and 405 nm light sources commonly used in modern dental 3D printers [60]. TPO is banned from EU cosmetics as a reproductive toxicant (September 2025), prompting regulatory‐driven shifts toward TPO‐L and BAPO in dental resins [61].

#### 4.1.3 Fillers

Although many 3D‐printed resins are unfilled, commercial dental crown materials for vat polymerization contain 3–60 wt% filler [54, 63], while denture base resins have been shown to incorporate 5–20 wt% filler [66]. The incorporation of filler into 3D‐printed resins has been shown to increase flexural strength, modulus, and hardness [54, 64]. However, the strengthening effect does not increase indefinitely. For example, an experimental resin demonstrated no further strength improvement beyond 40 wt% filler, and a commercial resin with 38 wt% filler exhibited greater strength than one with 50 wt% [67]. To ensure fillers remain evenly dispersed, manufacturers recommend mixing resins prior to printing. Microcomputed tomography (micro‐CT) analyses confirm that filler particles are distributed throughout printed objects, though particles align along printing layers and some particles tend to agglomerate into clusters [68]. The limitation to increasing filler loading in 3D‐printed materials is the associated increase in resin viscosity, which can complicate the printing process [62, 64]. For instance, raising filler content from 40 to 50 wt% increased viscosity by more than 50% [64]. Additionally, high filler loading can reduce light penetration, slowing photopolymerization [62].

### 4.2 Printed Ceramics

Commercially available systems are now capable of printing zirconia and lithium disilicate [69, 70]. Early evaluations of printed zirconia showed slightly lower strength and accuracy compared to milled zirconia, though still within clinically acceptable limits [71, 72, 73]. More recently, commercial grades of printed zirconia demonstrated higher strength than milled zirconia [21]. For lithium disilicate, studies have reported flexural strengths exceeding 300 MPa [69, 74, 75], highlighting its potential for functional dental restorations. The translucency of printed zirconia is significantly less than that of milled zirconia, which has been attributed to the presence of microporosities within the printed zirconia [76, 77].

## 5 Printed Splints

Compared with milling, 3D printing splints is generally more economical and allows the simultaneous production of multiple splints. Printed splints are available in different consistencies, often described as soft versus hard or flexible versus firm. Although flexible splints have a modulus 5–50 times lower than firm printed or milled splints [78], in clinical use they are still perceived as relatively rigid.

### 5.1 Properties

Review articles have reported that printed splints have inferior accuracy [79, 80] and mechanical properties [81, 82] relative to milled splints. Several studies reported lower flexural strength of 3D‐printed relative to alternatives [78, 83, 84, 85, 86, 87], with higher strength for firm printed splints than flexible ones [78]. However, reporting strength in printed splints can be misleading, since they often reach maximum stress without fracturing, suggesting that alternative properties may be more informative [78, 83, 84]. For fracture toughness, flexible 3D‐printed materials outperformed firm 3D‐printed resins, behaving more similarly to milled and heat‐polymerized materials [78]. This characteristic may be particularly relevant in preventing fractures in thin or sharp areas of splints. Furthermore, flexible printed materials demonstrated superior impact strength compared with firm printed or milled splints [78], implying better survival if dropped or subjected to sudden force.

A systematic review of laboratory studies concluded that heat‐polymerized, milled, and 3D‐printed splints demonstrate similar wear resistance, whereas thermoformed devices exhibit significantly worse wear resistance [88]. Individual studies vary; some report equivalent wear resistance for 3D‐printed and conventional devices [89, 90, 91], while others found significantly worse wear resistance in 3D‐printed devices [92, 93, 94]. In a study comparing firm and flexible printed splints, firm printed and milled materials exhibited the greatest wear resistance, while flexible printed materials showed the highest wear, exceeding even that of thermoformed resins [95].

In summary, flexible printed splints may offer advantages in fracture resistance relative to firm printed splints, whereas firm printed splints offer greater wear resistance. Flexible printed splints have toughness and impact resistance properties that suggest at least equivocal fracture resistance performance as milled splints [78]. Based on laboratory data, however, flexible printed splints would be expected to undergo more wear than milled and thermoformed splints [95]. A 3‐month pilot trial involving 47 patients found no significant differences between milled and flexible printed splints in terms of patient satisfaction, complication rates, or wear [96]; however, longer‐term clinical trials are lacking. Properties are summarized in Table 4.

**TABLE 4:** Comparison of clinically relevant properties of printed splints, crowns and dentures.

Material	Property	Comparison with milled/conventional materials	Clinical implications
Printed splints	Accuracy	Less accurate than milled or conventional fabricated splints [79, 80] yet clinically acceptable [79].	Suggests clinical acceptability for fit.
	Strength	Lower strength than milled or conventional [78, 83, 84, 85, 86, 87] but do not fracture during testing.	Flexible printed splints should be less likely to fracture if dropped than other types of splints. Firm printed splints are more susceptible to fracture in thin or sharp areas than flexible printed splints.
	Impact strength	Flexible printed splints have higher impact strength than milled and conventional splints. Flexible printed splints have higher impact strength than firm printed splints [78].	
	Fracture toughness	Flexible printed splints have higher fracture toughness than firm printed splints [78].	
	Modulus	Flexible printed splints are significantly lower modulus than firm printed splints which are slightly lower modulus than milled and conventional splints; Printed splints become more flexible in warm water [78].	Firm printed splints should have similar flexibility as milled and conventional splints whereas flexible splints are more flexible. Placing printed splints in warm water increases their flexibility.
	Wear resistance	Firm printed and milled splints have highest wear resistance, thermoformed intermediate and flexible printed splints the least [95].	Flexible printed splints may need to be replaced more frequently due to wear.
Printed crowns (resin‐based)	Accuracy	Internal fit, marginal fit, and trueness are generally comparable to those of milled restorations [5, 6, 7].	Suggests clinical acceptability for fit.
	Strength	Lower flexural strength than milled composite and ceramic materials [97, 98].	Occlusal thickness should be increased relative to milled composite and ceramic materials to prevent fracture. Should not be used in high stress indication such as posterior fixed partial denture.
	Fatigue resistance	Comparable to lithium disilicate [99, 100].	
	Modulus	Slightly lower modulus than milled composite and significantly lower modulus than ceramics [54, 101].	Flexing during occlusal loading may place stress at bond.
	Wear resistance	Less than milled composite [102, 103], mixed results compared to lithium disilicate [104, 105]. Clinical trial reported excessive wear [106]; material dependent [107]	Excessive wear may limit clinical survival of printed crowns. Some higher‐filled crown materials may be more wear resistant.
Printed crowns (ceramic)	Accuracy	Lower than milled ceramic yet clinically acceptable [71].	Suggests clinical acceptability for fit.
	Strength	Currently similar or higher than milled ceramic [21, 72, 73]	Suggests clinical acceptability for resistance to fracture.
	Translucency	Significantly less than milled ceramic [76, 77].	Should not be used for esthetic situations or would require veneering porcelain.
Printed dentures (base)	Accuracy	Occlusal fit, intaglio accuracy, and cameo accuracy inferior to milled dentures but similar or better than conventional dentures [8, 9]	Suggests clinical acceptability for fit.
	Strength and fracture toughness	Currently similar or higher than milled denture base [66, 108]; material dependent [66, 109]	Suggests clinical acceptability for resistance to fracture if a material with sufficient strength is selected.
	Translucency	Material dependent [66]	Should be aware of translucency of selected material, particularly if intending to hide components.
Printed dentures (teeth)	Wear resistance	Can be comparable or better than prefabricated or milled teeth [110, 111]; material dependent [112].	Suggests clinical acceptability if a material with sufficient wear resistance is selected.

### 5.2 Postprocessing

Postprocessing consists of rinsing (postrinsing) the splint after printing to remove uncured resin and subsequently curing it (postcuring) to maximize resin conversion. Proper postprocessing is essential not only for achieving optimal mechanical properties but also for minimizing residual monomer release and reducing cytotoxicity [113]. Postprocessing protocols are summarized in Table 5.

**TABLE 5:** Postprocessing considerations for 3D‐printed splints, crowns, and dentures.

	Splints	Crowns	Denture bases^a^
Cleaning	Typically rinsed in isopropyl alcohol (IPA); alternative cleaning solutions may also be acceptable. IPA rinsing times between 5 min and 1 h yield comparable surface texture, mechanical properties, and biocompatibility, whereas prolonged rinsing (e.g., ≥ 12 h) adversely affects surface integrity and strength [114].	Commonly rinsed in 90%–99% IPA [115]. Extended rinsing (> 10–60 s) reduces flexural strength [115, 116, 117] and produces a frosted surface appearance [118], particularly, in highly filled materials. TPM may be an acceptable alternative [115].	*Vat polymerization*: Flexural strength and stiffness are largely maintained after postprinting washing times of up to 90 min in IPA [119]. *MultiJet*: Wax supports are frozen, thermally removed in a furnace, and cleaned using pressurized pumice [120]. *PolyJet*: Gel‐like supports are removed with pressurized water, often followed by soda‐water soaking [121].
Curing	Increased curing time and temperature improve degree of conversion and mechanical properties [122]. Oxygen‐inhibition control using glycerin improves stain resistance [123] and nitrogen improves surface conversion [124]; short‐term storage of cured splints in water at 100°C (≈5 min) may reduce cytotoxic components [125, 126].	Mechanical properties improve with higher curing energy and elevated temperature [115, 127]. No significant differences have been reported between 385 and 405 nm curing, whereas 460 nm curing is inferior [128]. Nitrogen curing offers minimal benefit [129]. Polywave LCUs may be used as alternatives to dedicated curing chambers [130].	*Vat polymerization*: Curing under glycerin enhances mechanical strength [131] and reduces water sorption and solubility [132]. *MultiJet and PolyJet*: Postcuring is generally not required unless photocuring required for color stability
Surface finishing	Laboratory polishing produces low surface roughness and high translucency [123, 133]. Resin surface coatings can achieve comparable smoothness but require glycerin‐assisted curing to prevent staining [123].	Low‐filled materials polish effectively with goat‐hair polishers, whereas highly filled materials require diamond polishing systems [134]. Varnish coatings provide durable smooth surfaces with thicknesses of approximately 40–180 μm [134, 135].	Conventional polishing with pumice and polishing paste is standard [136]. Nanoparticle‐reinforced glazes may improve hardness, strength, and microbial resistance [132, 137, 138], although reported effects on surface roughness are inconsistent [137, 138].
Bonding	Not applicable	Airborne‐particle abrasion improves bond strength [139]. Silane application to the intaglio surface is optional, whereas application of an adhesive resin is critical [118, 140].	Critical for split‐denture workflows. Airborne‐particle abrasion improves bonding [141]; both MMA‐based primers and dimethacrylate adhesive systems have demonstrated effectiveness [141, 142, 143].

^a^ Denture teeth resin is often the same or similar to crown resin.

Manufacturers typically recommend postrinsing printed splints in isopropyl alcohol, however, some alcohol‐free alternative rinses may yield comparable mechanical and biocompatibility outcomes [144]. Rinsing times of 5–60 min are acceptable as they produce similar surface and mechanical properties, however prolonged rinsing (e.g., 12 h) adversely affects surface quality and strength [114].

Commercial resins should be postcured using their recommended curing profiles in light‐curing units (LCUs) validated by the resin manufacturer as postcuring time, temperature, and light‐curing machine irradiance all increase degree of resin conversion [122]. Despite postcuring, residual monomer release has still been observed, with cytotoxic effects on tongue epithelial cells in laboratory tests [125]. To mitigate this, cured splints may be stored at 100°C for 5 min to encourage leaching of cytotoxic components [125, 126].

An additional consideration is the removal of the oxygen inhibition layer on the surface of prints which could worsen external staining and surface properties. Postcuring splints in glycerin is essential for preventing staining, while vacuum curing showed no benefit in reducing staining [123, 145]. Curing in nitrogen can improve conversion and hardness of splints but not their strength [124]. Some printed splint materials yellow after printing or postcuring caused by benzoyl‐type radicals from the photoinitiator forming colored secondary products (chromophores) that gradually oxidize and bleach [146]. As bleaching is expedited by oxygen, water, and heat, curing in the presence of oxygen or a warm‐water hold after curing may help reduce the yellow tint [146].

### 5.3 Polishing and Resin Coating

After postprocessing, printed splints require removal of supports and smoothing of the external surface. Using a laboratory polishing wheel with pumice and polishing compound effectively achieves low roughness and high translucency [123, 133]. An alternative is resin coating (so‐called “candy coating”), where a thin layer of the same uncured resin is applied to the surface and cured. This creates a smooth finish that is comparable to laboratory polishing [104, 123], but a final cure in glycerin is necessary to prevent staining [123].

Another approach is to print directly on an optical polish tank, which incorporates an intensity‐controlling film to minimize voxel‐related staircase artifacts in DLP [123]. This technique enhances surface smoothness and translucency but optical polish tanks currently studied did not achieve the same roughness values in in vitro testing as polishing or resin coating [123].

## 6 Printed Crowns

In 2023, the American Dental Association's Council on Dental Benefit Programs revised the definition of “porcelain/ceramic” to include “materials containing predominantly inorganic refractory compounds,” removing any requirement for a specific fabrication method (e.g., milling or pressing). This change allows “highly filled” 3D‐printed crown materials to be classified as definitive ceramic restorations [147]. The resins used for crowns may overlap with those used for printed denture teeth and interim implant‐supported hybrid prostheses due to similar performance requirements, namely tooth‐colored esthetics, strength, and wear resistance. In cross‐arch applications, however, toughness may be prioritized to dissipate stresses over long spans and avoid catastrophic fracture at stress concentrators, even at some expense of stiffness. By contrast, single‐unit restorations benefit from higher stiffness to minimize micromovement under load [54].

### 6.1 Properties

Printed crown materials, including those indicated for definitive use, demonstrate lower flexural strength than milled composite and ceramic materials [97, 98]. Reported flexural strength values for definitive printed crowns range from 108 to 144 MPa in three‐point bending [54, 101, 148] and 149–185 MPa in biaxial testing [149], compared with 184 and 286 MPa for milled composites and 300 and 746 MPa for lithium disilicate, respectively [54, 149]. Despite these substantially lower strength values, manufacturer‐recommended minimum thicknesses for posterior printed crowns typically range from 1.0 to 1.5 mm [150, 151]. A recent laboratory study evaluating a permanent printed crown material with approximately 25% filler content demonstrated increased strength with increasing occlusal thickness from 1.25 to 2.0 mm [152]. The authors recommended a thickness of 2.0 mm to accommodate clenching forces [152]. When extended to higher‐stress applications, such as posterior fixed partial dentures, printed crown materials exhibited a 3‐year mechanical clinical survival rate of only 40.7%, indicating that these materials would not be indicated for posterior fixed partial dentures [153].

The comparatively lower elastic moduli of printed crown materials (2–7 GPa) relative to milled composites (12 GPa) and lithium disilicate (79 GPa) [54, 101] confer increased toughness [54], which may contribute to improved fatigue performance. Consistent with this hypothesis, 3D‐printed occlusal coverage restorations bonded to tooth or resin dies have demonstrated fatigue survival comparable to lithium disilicate [99, 100]. Accordingly, printed crown materials have been advocated for thin occlusal restorations intended for short‐term alteration of the vertical dimension of occlusion [106]. In a 2‐year clinical trial of a moderately filled printed crown material, an 84% survival rate was reported; however, 35% of restorations exhibited more than 0.5 mm of maximum vertical height loss [106]. These clinical findings align with laboratory studies demonstrating greater wear of printed crown materials compared with milled or conventional composites [102, 103], however, mixed outcomes have been reported of the wear of printed crowns relative to lithium disilicate [104, 105]. One laboratory study reported greater wear resistance for a 50% filled printed crown material compared with two unfilled printed materials, indicating that wear of printed crowns is material dependent [107]. Properties are summarized in Table 4.

### 6.2 Postprocessing

Isopropyl alcohol (90%–99%) is the most commonly used postrinsing solvent for printed crown resins [115]. Tripropylene glycol monomethyl ether has been proposed as a lower‐flammability, reusable alternative, with studies reporting either comparable strength or an approximately 10% reduction relative to isopropyl alcohol [115]. Other rinse agents, including ethanol, acetone, bioethyl alcohol, and water‐miscible formulations, generally reduce flexural strength [115]. Prolonged rinsing (> 10–60 s) should be avoided as it has been associated with reduced strength [115, 116, 117], likely due to extraction of subsurface unpolymerized resin and subsequent shrinkage‐related surface defects during postcuring [116]; however, a low‐filled material (3%) showed increased strength with longer rinsing [115, 154, 155]. Manufacturers of highly filled resins recommend only brief immersion (e.g., ≤ 5 s) [151], as extended soaking may also produce surface frosting from filler residue [118].

Postcuring effectiveness depends on delivered energy (power × time), with longer curing times [115] and higher‐power units [127] generally enhancing mechanical properties. High‐power curing units can achieve similar outcomes with shorter exposures [156], and curing device selection exerts a greater influence on mechanical properties than printer choice [157]. Comparable properties are achieved with 385‐ and 405‐nm light, whereas 460‐nm curing yields inferior results [128]. Elevated curing temperatures further enhance mechanical performance [158], even in devices without active heating [156]. Nitrogen atmospheres do not significantly improve properties [129]. An alternative to a desktop curing chamber is postcuring with a dental polywave LCU. Using a polywave LED LCU at 3200 mW/cm^2^, four 3‐s exposures per surface produced hardness and strength comparable to a desktop device [130]. Shorter LCU curing times yielded inferior properties, and adding an LCU cure after a full desktop cure provided no additional benefit [130, 159].

### 6.3 Polishing and Varnishing

Printed crowns are generally less filled and softer than direct composites, requiring specific polishing approaches. For softer printed crown materials with a filler content of 33 wt% or less, goat‐hair polishers are an effective choice [134, 135, 160]. In contrast, for materials with a higher filler content, diamond‐impregnated rubber polishers have proven more efficient [134]. An alternative to mechanical polishing is the application of a light‐cured, unfilled varnish. This method is often easier and faster than polishing and has been shown to produce surface roughness equivalent to polished restorations [134, 160]. Importantly, the smooth surface achieved with varnish can be maintained even after simulated toothbrushing in laboratory conditions [134, 135]. The varnish layer typically ranges from 40 to 180 μm in thickness [134, 160], therefore, omitting glaze application on occlusal surfaces may be advantageous to avoid altering occlusion.

### 6.4 Bonding

Bonding procedures for printed resin composite crown materials should begin with alumina airborne‐particle abrasion, as this method has been shown to improve bond strength [139]. Increasing abrasion pressure from 1 to 3 bar can enhance bond strength but also increases the risk of fracturing within the crown material [161]. A universal adhesive should be applied to the crown intaglio surface prior to resin cementation [118, 140]; prior silane application can be performed but does not significantly improve the bond [118].

## 7 Printed Dentures

Printed dentures may be fabricated following either a workflow in which denture teeth and base are printed as a single prosthesis (monolithic dentures) or one in which the denture teeth and base are printed separately (split dentures). The primary advantage of a monolithic denture is the reduced risk of occlusal discrepancies and adhesive tooth debonding, as the prosthesis is fabricated as a single unit without an assembly process combining separately manufactured teeth and base materials [162].

### 7.1 Monolithic Dentures

Single‐shade printed monolithic dentures may be produced by vat printing the entire denture with a denture tooth material and adding specialized resin stains or tissue‐shaded composites to the gingival aspect of the denture [136]. Characterization composite may be applied following sandblasting and adhesive application [118, 139] or directly to the printed denture before postcuring without the use of an adhesive [136]; insufficient evidence is present to determine the best clinical protocol. Characterization composite has been shown to achieve a higher bond to printed denture base than milled denture base materials [163].

Another method of fabricating a monolithic printed denture is printing the gingival‐ and tooth‐portions at the same time using material jet printing. Material jetting is an additive manufacturing technique in which UV‐curable photopolymer resins are deposited in fine layers by a printhead moving along the *X*‐ and *Y*‐axes, with each layer immediately polymerized by integrated ultraviolet light before incremental movement along the *Z*‐axis [120, 121, 142]. This approach enables high‐resolution fabrication, with layer thicknesses as low as 16 μm, and allows simultaneous printing of gingival‐ and tooth‐colored resins within a single build [120]. A limitation of material jetting is material wastage, as routine purging of printheads to prevent nozzle clogging results in loss of unused, nonrecyclable resin requiring regulated disposal [164].

Material jetting systems are primarily categorized as MultiJet (e.g., ProJet MJP 2500, 3D Systems) and PolyJet (e.g., J5 DentaJet, Stratasys, Rehovot, Israel). Both systems allow multi‐shade fabrication, however PolyJet printers simultaneously dispenses and mixes multiple liquid photopolymer resins, enabling gradients in color, translucency, and mechanical properties within a single print [142, 165]. The technologies also vary in the postprocessing, MultiJet printers use a paraffin‐based wax support material removed by freezing, thermal processing, and pumice cleaning [120], whereas PolyJet technology uses a gel‐like support material that is coprinted and subsequently removed using pressurized water and alkaline soaking [121].

### 7.2 Split Dentures

In the split denture approach, the denture base is printed via vat polymerization and the teeth may be conventionally manufactured acrylic teeth or separately printed denture teeth [121, 136]. A challenge with split dentures is ensuring adhesion between the teeth and base. Printed split dentures have shown similar fatigue survival as conventional dentures fabricated via the compression‐molding technique; however, fractures at the base‐tooth interface occurred more frequently in printed dentures [166]. The teeth may be secured to the base by lining the socket areas of the base with either uncured denture base resin or a product‐specific adhesive, after which the assembly is polymerized in a curing chamber [121, 136]. Initial mechanical roughening of the printed denture base with alumina abrasion improves bond strength [141]. There is conflicting evidence regarding the optimal chemical approach for bonding denture teeth to printed denture bases. Both methacrylate‐based primers and dimethacrylate‐containing adhesives have demonstrated clinically acceptable bonding performance, and their effectiveness appears to be material‐ and system‐dependent [141, 142, 143]. Dimethacrylate‐based bonding systems may provide higher bond strength in some workflows [142], whereas MMA‐based primers have shown reliable bonding in others [143]. As a result, clinicians are advised to prioritize surface roughening followed by use of the bonding protocol recommended for the specific printed denture base system.

### 7.3 Properties

A recent meta‐analysis concluded that 3D‐printed denture base materials exhibit lower flexural strength and impact strength compared with milled and conventionally processed bases [109]. However, recent studies indicate that several contemporary printed materials can achieve equal or greater strength and fracture toughness than both milled and conventional materials [66, 108]. Material‐jetted denture bases demonstrated comparable strength to vat‐polymerized resins, but exhibited lower flexibility and fracture toughness than high‐performing vat‐polymerized formulations [66]. In terms of optical properties, some vat‐polymerized denture bases display higher translucency than heat‐cured bases, which may increase the risk of attachment component show‐through, while others match the translucency of heat‐cured materials [66]. These results demonstrate that printed denture base material may have mechanical and optical properties similar to milled and conventionally fabricated denture bases; however, properties are material dependent.

In vitro wear testing of printed denture teeth opposing zirconia exhibit wear resistance better than prefabricated teeth and comparable to or better than milled denture teeth [110, 111]. Wear testing opposing steatite antagonists (a softer enamel analog) revealed one printed denture tooth material with similar wear resistance as a prefabricated denture tooth, but two other printed teeth with inferior wear resistance [112]. These results demonstrate clinical feasibility of some printed denture teeth relative to commonly used alternatives. Properties are summarized in Table 4.

### 7.4 Postprocessing

Similar to printed splint materials, 3D‐printed denture base resins are generally low in inorganic filler content. Consequently, their flexural strength and stiffness are largely maintained even after extended postprinting washing times of up to 90 min in IPA [119]. In addition, postcuring denture base resins under glycerin has been shown to further enhance mechanical strength while reducing water sorption and solubility [131, 132].

### 7.5 Polishing and Glazing

Polishing of 3D‐printed denture base materials is typically performed using a polishing wheel with pumice followed by a polishing paste [136]. Printed dentures can also be surface‐sealed with nanoparticle‐reinforced glazes, which have been reported to increase surface hardness, enhance strength, decrease solubility, decrease sorption, and reduce *C. albicans* colonization [132, 137, 138]. Evidence regarding their effect on surface roughness is mixed [137, 138]. Laboratory studies have also demonstrated that multi‐step, silicon carbide–impregnated polishers can restore the surface roughness of denture bases, previously adjusted with acrylic burs [167, 168].

### 7.6 Maintenance

When relined with rigid materials, 3D‐printed denture bases perform comparably to milled and conventional bases because the solvents and monomers in hard reline resins can penetrate residual monomer sites and form an interpenetrating polymer network despite the printed base's high cross‐link density. In contrast, soft liners adhere less effectively to printed bases than to conventional PMMA, as the highly cross‐linked, photo‐polymerized surface resists softening from soft liner monomer which limits mechanical interlocking [169]. Guidance on optimal repair protocols is limited due to heterogeneity among studies, though resin composite is often recommended [170]. Surface roughening has been shown to enhance bonding for both relining and repair [169]. For hygiene, immersion in Listerine mouthrinse for 20 min has been reported to reduce *C. albicans* growth and preserve mechanical properties more effectively than effervescent tablets or dilute bleach [171].

## 8 Conclusion

3D printing is now a clinically viable manufacturing approach for splints, crowns, and dentures, but outcomes remain highly material‐ and workflow‐dependent. Clinicians and laboratories must use validated printer‐resin systems and adhere to manufacturer‐specified postprocessing. For splints, flexible formulations may better resist fracture while firmer formulations may better resist wear. For crowns, their lower strength and higher wear potential relative to milled composites and ceramics emphasize the importance of appropriate thickness and case selection, and they are unsuitable for high‐stress multiunit posterior applications. Printed ceramics show promising strength, but common adoption will require improvements in processing efficiency and optical properties. For dentures, both monolithic and split workflows are feasible; however, clinical success depends on selection of materials and workflows to minimize potential failure modes (e.g., tooth‐base debonding, loss of characterization stains, denture base show‐through, impact fracture). Overall, 3D printing offers advantages in cost and manufacturing efficiency, but clinicians should be diligent with material selection and fabrication workflow.

## 9 Limitations and Future Perspectives

This review is limited by the heterogeneity of available studies, including variability in printer models, material formulations, printing parameters, postprocessing protocols, and testing methodologies, which complicates direct comparison across investigations. Much of the current evidence is derived from in vitro studies, and long‐term clinical data, particularly for definitive printed crowns and dentures, remain sparse.

Several areas of future laboratory research could be considered to bridge knowledge gaps and optimize clinical workflows: performing fatigue testing of printed crowns of different thicknesses relative to a ceramic control to better determine a recommended occlusal thickness, comparing contemporary optical polish tanks for printed splint materials, optimizing a protocol for adding characterization stains to single‐shade printed dentures and bonding teeth to printed denture bases, and evaluating materials for improving soft relines to printed dentures.

## Funding

The authors have nothing to report.

## Conflicts of Interest

Dr. Lawson has received grants and honorarium from Ivoclar Vivadent, Dentsply, Kulzer, and GC. Dr. Rocha has received grants and honorarium from Ivoclar and Dentsply.

## Data Availability

Data sharing not applicable to this article as no datasets were generated or analysed during the current study.
